# Supplementary material for: Integrated Analysis of the Lung Microbiome and Metabolome Reveals Associations Between Amino Acid Metabolism and Pulmonary Fibrosis in a Bleomycin-Induced Mouse Model
Source: Int J Mol Sci. 2026 Jun 30;27(13):5895. doi: 10.3390/ijms27135895 (PMC13362081; doi:10.3390/ijms27135895)
Supplement: Supplementary file 1 [file ijms-27-05895-s001.zip › result/4.MetDiffAnalysis/B_28d.vs.C_28d/B_28d.vs.C_28d_all_chord.pdf]

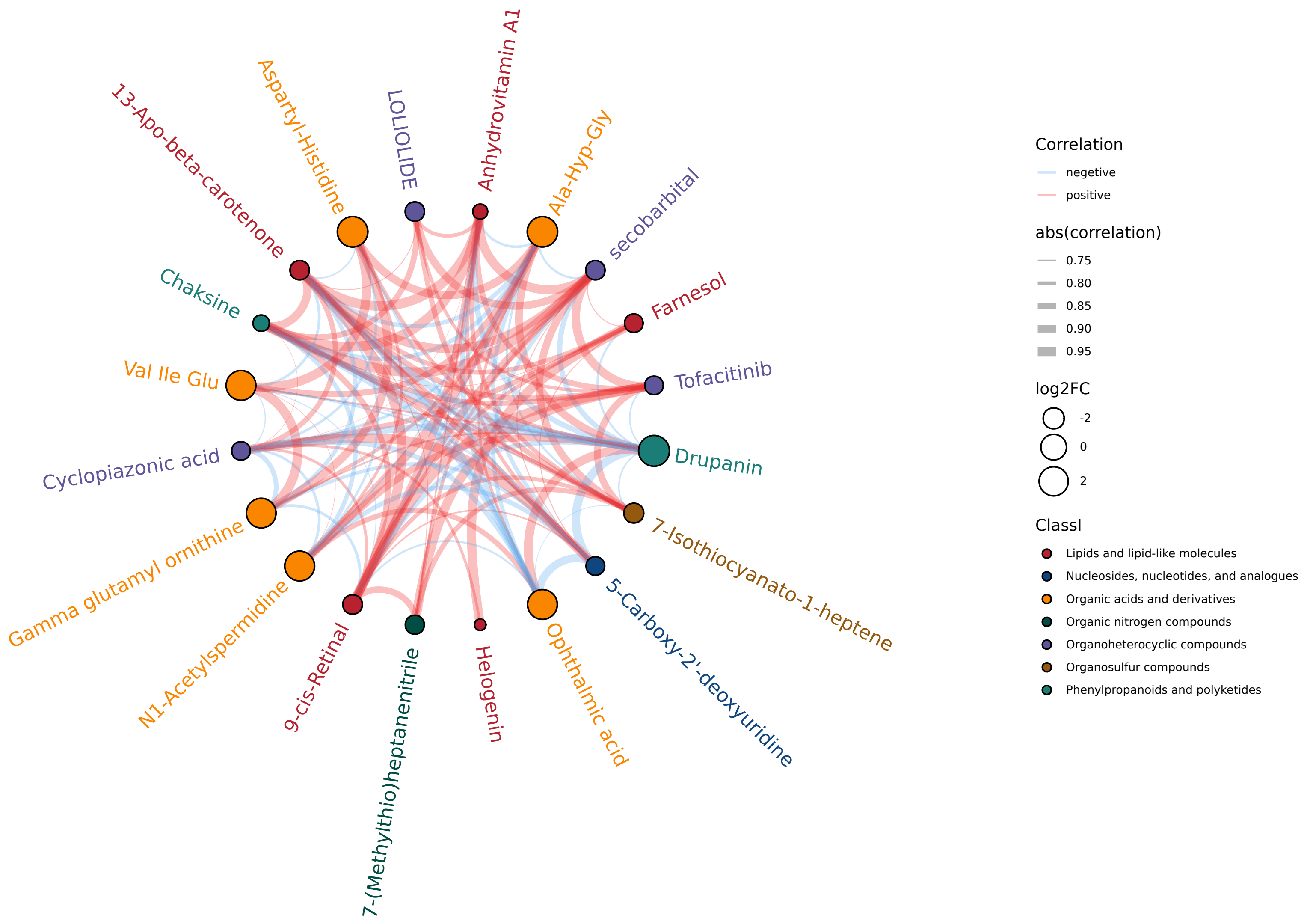

13-Apo-beta-carotenone
Aspartyl-Histidine
LOLIOLIDE
Anhydrovitamin A1
Ala-Hyp-Gly
secobarbital
Farnesol
Tofacitinib
Drupanin
7-Isothiocyanato-1-heptene
5-Carboxy-2'-deoxyuridine
Ophthalmic acid
Helogenin
7-(Methylthio)heptanenitrile
9-cis-Retinal
N1-Acetylspermidine
Gamma glutamyl ornithine
Cyclopiazonic acid
Val Ile Glu
Chaksine
Correlation
negetive
positive
abs(correlation)
0.75
0.80
0.85
0.90
0.95
log2FC
-2
0
2
ClassI
Lipids and lipid-like molecules
Nucleosides, nucleotides, and analogues
Organic acids and derivatives
Organic nitrogen compounds
Organoheterocyclic compounds
Organosulfur compounds
Phenylpropanoids and polyketides
